# Evaluating conserved domains and motifs of decapod gonadotropin-releasing hormone G protein-coupled receptor superfamily

**DOI:** 10.3389/fendo.2024.1348465

**Published:** 2024-02-20

**Authors:** Sean J. Buckley, Tuan Viet Nguyen, Scott F. Cummins, Abigail Elizur, Quinn P. Fitzgibbon, Gregory S. Smith, Donald L. Mykles, Tomer Ventura

**Affiliations:** ^1^ Centre for Bioinnovation, University of the Sunshine Coast, Maroochydore, QLD, Australia; ^2^ School of Science, Technology and Engineering, University of the Sunshine Coast, Maroochydore, QLD, Australia; ^3^ Centre for AgriBioscience, Agriculture Victoria, Bundoora, VIC, Australia; ^4^ Institute for Marine and Antarctic Studies (IMAS), University of Tasmania, Hobart, TAS, Australia; ^5^ Department of Biology, Colorado State University, Fort Collins, CO, United States; ^6^ University of California-Davis Bodega Marine Laboratory, Bodega Bay, CA, United States

**Keywords:** GPCR, evolutionary history, GnRHR, conserved motifs, decapoda

## Abstract

G protein-coupled receptors (GPCRs) are an ancient family of signal transducers that are both abundant and consequential in metazoan endocrinology. The evolutionary history and function of the GPCRs of the decapod superfamilies of gonadotropin-releasing hormone (GnRH) are yet to be fully elucidated. As part of which, the use of traditional phylogenetics and the recycling of a diminutive set of mis-annotated databases has proven insufficient. To address this, we have collated and revised eight existing and three novel GPCR repertoires for GnRH of decapod species. We developed a novel bioinformatic workflow that included clustering analysis to capture likely GnRH receptor-like proteins, followed by phylogenetic analysis of the seven transmembrane-spanning domains. A high degree of conservation of the sequences and topology of the domains and motifs allowed the identification of species-specific variation (up to ~70%, especially in the extracellular loops) that is thought to be influential to ligand-binding and function. Given the key functional role of the DRY motif across GPCRs, the classification of receptors based on the variation of this motif can be universally applied to resolve cryptic GPCR families, as was achieved in this work. Our results contribute to the resolution of the evolutionary history of invertebrate GnRH receptors and inform the design of bioassays in their deorphanization and functional annotation.

## 1 Introduction

The decapods are an order of crustaceans that constitute an important global source of dietary protein for humans and are being harvested in increasingly large numbers using aquaculture facilities (1). While finite in number, the members of the decapod order are hyper-diverse in biology (for example, freshwater and salt water crabs, shrimps, lobsters and crayfish) (2), which represents a real challenge in understanding decapod biology. Moreover, a better understanding of the endocrinology that controls the decapod growth and fecundity is a major goal of crustacean aquaculture biologists (3, 4). To date, neuropeptides of the decapod gonadotropin-releasing hormone (GnRH)-like superfamily have been implicated in the reproduction-related maturation of ovaries and proliferation of oocytes (5), raising the likelihood that the characterization of the GnRH-like receptors and their role in signal transduction promises the development of important future aquaculture technologies.

G protein-coupled receptors (GPCRs) are a family of ancient cell membrane spanning signal transducers that are found across the eukaryotic tree of life (including crustaceans and humans), making them the key target for medical drugs (6, 7). Despite their ancestral origin, GPCRs display remarkably conserved topological domains that are defined by their relationship to the hydrophobic phospholipid bi-layered cell membrane. Canonically, these include the extracellular N-terminus, intracellular C-terminus, seven transmembrane spans (7TMs), three extracellular loops (ECLs) and three intracellular loops (ICLs) (8). While the N-terminus and ECLs compose the extracellular ligand binding sites, the C-terminus and ICLs compose the intracellular binding site(s) of the titular associated G protein (9). Members of decapod GnRH-like receptors are GPCRs (10).

The identification of the GnRH decapeptide in the hypothalamo-pituitary regulation of reproduction was a major step in both basic and clinical reproductive endocrinology, winning Roger Guillemin and Andrew Schally the 1977 Nobel Prize in Physiology and/or Medicine. This decapeptide was found to date back 400 million of years in evolution, raising the question of what this peptide function was in the absence of a hypothalamus or pituitary {Kochman, 2012 #2894}.

The ancestral history of the receptors of the superfamily of invertebrate GnRH-like neuropeptides is unresolved (5), undergoing proposed nomenclature reassignment (11), and requires further evidence that contributes to its resolution. By interpolation, the same holds true for the crustacean counterparts. The functional annotation of orphan GPCRs is resource-intensive and not trivial. In fact, few neuropeptide receptors have been deorphanized in crustaceans (12–14), lagging behind functional neuropeptide GPCR deorphanization in insects (15). New databases which were recently developed including CrustyBase (16) and CrusTome (17) provide ample opportunity to bridge this gap by exploring expression patterns and phylogenetic analyses of genes across multiple pancrustacean species. Therefore, any measure that appreciably narrows the focus by reducing the relevant search space is of potential worth. To date, the bioinformatic annotation of crustacean GPCRs using Basic Local Alignment Search Tool (BLAST) databases and traditional phylogenetic analysis has proven insufficient in the description of GnRH-like GPCRs. Understanding any species-specific sequence variation and the congruence of conserved, function-related GPCR domains and motifs, serves to better define the phylogeny of GnRH-like receptors and contextualize their functional annotation studies.

Within the context of the conserved topological domains, GPCRs have been observed to possess several highly conserved and function-related aa motifs including ‘DRY’, ‘CWxP’ and ‘NPxxY’, which are located at the union of transmembrane domain 3 (TM3) and ICL2, within TM6, and with TM7, respectively. The DRY motif is thought to create a network of interactions (including ‘salt bridges’ or ‘ionic locks’) that keep the receptors in the inactive conformation, ultimately playing a crucial role in the activation of the receptor upon ligand binding (18). The binding of the G protein to the GPCR has also been observed to occur in a binding pocket that is topologically and functionally related to the DRY motif locus (19). Similarly, conserved CWxP and NPxxY motifs have been implicated in GPCR activation, and ligand and G protein binding (20).

In this study, we describe well-resolved evolutionary histories of decapod GnRH-like receptors. We annotate clades of receptors of GnRH (GnRHR), corazonin (CrzR), red pigment concentrating hormone (RPCHR), adipokinetic hormone/corazonin-related peptide (ACPR1 and ACPR2), within the context of the closely-related receptors of vasopressin (VR) and crustacean cardioactive peptide (CCAPR).

## 2 Methods

### 2.1 Standardization of GPCR-encoding transcripts

The putative GPCR-encoding transcripts of eleven decapod species (including crab, shrimp, crayfish and lobster from both freshwater and seawater) were compiled into a single catalogue using the contemporary methods described below. Previously published GPCRomes (i.e., all proteins identified as putative GPCRs of an organism) that have been incorporated into this study include: *Procambarus clarkii* (21), *Sagmariasus verreauxi* (10), *Nephrops norvegicus* (22), *Carcinus maenas* (23), *Gecarcinus lateralis* (24) and *Penaeus monodon* (25). Publicly available transcriptomic reads accessed from NCBI Transcriptome Sequence Assembly (TSA) database include: *Homarus americanus*, *Cherax quadricarinatus* and *Eriochier sinensis*. Novel GPCRomes were generated for *Panulirus ornatus*, *C. quadricarinatus* and *E. sinensis* and are presented as part of this study. A summary of the species, tissues, assembly technology, and origin of transcriptomic reads is summarized in **Table 1**.

**Table 1:** Summary of the original data used in this study.

Species	Tissues used for RNA-Seq	*De novo* assembler used	Accession numbers/References
*Cancer borealis (Cb)*	Nervous system (brain, abdominal nerve cord, cardiac ganglion and complete stomatogastric nervous system	CLC NGS Cell 5.0.1	GEFB00000000
*Carcinus maenas (Cm)*	Central nervous system	Trinity v2.0.6	GFXF00000000
*Cherax quadricarinatus (Cq)*	Heart, kidney, liver, nerve, testis	Trinity	HACB00000000
*Eriocheir sinensis (Es)*	Eyestalk	Trinity	GBZW00000000
*Gecarcinus lateralis (Gl)*	Y-organ across the molt	Trinity	(24)
*Homarus americanus (Ha)*	Eyestalk, brain	Trinity 2.0.6	GFUC00000000
*Nephrops norvegicus (Nn)*	Brain, thoracic ganglia, eyestalk, ovary, testis	Trinity	(22)
*Procambarus clarkii (Pc)*	Eyestalk, brain, hemocytes, gills, testis, ovary, hepatopancreas, heart, green gland, ventral ganglia, Y-organ, hypodermis, muscle	CLC NGS Cell 6.51	GBEV00000000
*Penaeus monodon (Pm)*	Brain, thoracic ganglia, eyestalk, ovary and antennal glands from previtellogenic and vitellogenic females	Trinity v2.4.0	(25)
*Panulirus ornatus (Po)*	Brain, thoracic ganglia	Trinity	(26–28) PRJNA903480, PRJNA761502, and PRJNA664650.
*Sagmariasus verreauxi (Sv)*	Five developmental stages throughout metamorphosis (phyllosoma and puerulus)	Trinity	(10)

### 2.2 Contemporary methods employed in the identification of the putative GPCR-encoding transcripts of *Panulirus ornatus*, *Eriocheir sinensis* and *Cherax quadricarinatus*

The sampling of tissue, RNA extraction, RNA quality assurance and RNA read sequencing for *P. ornatus* have been outlined previously (26–28). The resulting read sequences were subjected to the *de novo* assembly algorithm of CLC Genomics Workbench v9.5 (https://www.qiagenbioinformatics.com/) and cDNA was translated into the longest open reading frames (ORFs) using CLC. All ORFs were then screened against the Pfam database for predicted GPCR structural domains using the CLC platform with the Pfam module (v29). Search results displaying maximum E-values of 10^-3^ were extracted and iteratively used as a reference list. Additionally, a search was conducted on UniprotKB database using the terms “family: G protein coupled receptor 1 family and taxonomy: Arthropoda [6656]” for Rhodopsin receptors, and “family: G protein coupled receptor 2 family and taxonomy: Arthropoda [6656]” for Secretin receptors. Rhodopsin and Secretin reference BLAST databases were generated from these lists and a local BLASTp search was conducted to discriminate the class A (Rhodopsin-like) and class B (Secretin-like) decapod GPCRs. The N-terminus and C-terminus of all predicted GPCR-encoding transcripts were trimmed to also yield lists of 7TM domains (including ECLs and ICLs).

### 2.3 Novel bioinformatic workflow for the annotation of GPCRs

Predicted class A (Rhodopsin-like) decapod GPCR-encoding transcripts were then subjected to a bioinformatic workflow which was designed to assist in the classification and functional annotation of GPCRs by reducing the relevant search spaces. Key steps in the workflow included clustering analysis (incorporating deorphanized and predicted orthologues), and the determination of the topological GPCR domains and conserved motifs (that is DRY, CWxP, NPxxY). The relationships between the sequences of the function-related DRY motif locus and the phylogeny of the 7TM domains was then analyzed for predicted receptors of the GnRH-like superfamily of neuropeptides. The congruence and compartmentalization of species, conserved motifs and phylogenetic clades was then qualified collectively, allowing the annotation of receptors into clades.

Firstly, all available class A decapod GPCR ORFs were downloaded from the Interpro database using the search terms ‘Rhodopsin-like’ and ‘7TM’. A combined list of class A decapod GPCRs was compiled that incorporated the transcripts from Interpro, our eleven decapod species and selectively ‘seeded’ decapod GnRH-like receptor transcripts that have previously been functionally or bioinformatically annotated. Clustering analysis was then performed on the combined list using the CLANS2 algorithm (29) with a P-value of 1e-50 for 10,000 rounds in three dimensions. The resulting clusters were then collapsed to two dimensions for visualization. In brief, CLANS2 performs all-against-all BLAST searches in the three-dimensional space where clustering is performed using attractive forces proportional to the negative logarithm of the BLAST P-values, and a uniform repulsive force.

A list of the available functionally annotated receptors of the decapod superfamily of GnRH-like neuropeptides (that is, GnRH, corazonin, RPCH, ACP, CCAP and vasopressin) was compiled as seeds. The seeded transcripts included the functionally annotated CCAPR of *Scylla paramamosain* (Sp_CCAPR) (30), and corazonin receptor (Cm_CrzR: accession number MF974386) and RPCH receptor (Cm_RPCHR: accession number MF974387) of *Cm* (31), as well as the bioinformatically annotated vasopressin receptors (VRs) of *Portunus pelagicus* (Pp_VR: accession number MZ147830.1) and of *Litopenaeus vannamei* (Lv_VR: accession number ROT66533.1), and the ACP receptor of *Macrobrachium rosenbergii* (Mr_ACPR) (32). Transcripts that clustered with these seeds were extracted and considered putative GnRHR-like transcripts.

The candidate GnRHR-like transcripts were then subjected to the online machine-learning DeepTMHMM algorithm in the prediction of GPCR topological domains (including N-terminus, C-terminus, ECLs, ICLs, and 7TM) (33). Identification of these domains enabled the subsequent prediction of the highly conserved DRY, CWxP, and NPxxY motifs. The aa sequences of both the 7TM (including ECLs and ICLs) and the transcript ORFs of the GnRHR-like receptors were aligned using the MUSCLE algorithm (34) implemented in MEGA-X (35). Then neighbor joining phylogenetic trees were constructed (bootstrap values of 1,000) using Mega-X. The resulting phylogenetic clades that were constructed using both the 7TMs (including ECLs and ICLs) and the ORFs were compared and contrasted to assess the propriety of using either method in this study.

Snake plots were generated using Protter (wlab.ethz.ch/protter) (36) for the functionally annotated Cm_CrzR, Cm_RPCHR and Sp_CCAPR, and three transcripts from this catalogue (Gl_GPCR_A7, Po_GPCR_A146 and Cm_GPCR_A117) that respectively shared a high degree of sequence identity. Snake plots of the ACPR1 and ACPR2 clades were generated to compare the intra-clade and inter-clade similarities and differences, and especially the ECLs. All snake plots were augmented with the species, conserved GPCR motifs, and a color-coded annotation.

The aa sequences of the 7TM (including ECLs and ICLs) of the GnRHR-like transcripts were aligned and Neighbor Joining phylogenetic trees were constructed, as described above. The phylogenetic trees were also labeled with species and associated DRY motif sequence. The compartmentalization of labels and the strength of phylogenetic clades were analyzed and visually represented. In considering the two ACPR clades (grey and orange), that lacked a functionally annotated constituent, we identified the sequences of the conserved motifs of the two functionally annotated ACPR-like receptor variants, BNGR-A28 (accession number: NP_001127726.1) and BNGR-A29 (accession number: NP_001127745.1) of silkworm moth *Bombyx mori* (37). The sequences of the conserved DRY, CWxP, NPxxY motifs of all receptor transcripts were tabulated and sorted. The congruence and compartmentalization of species and conserved motifs was analyzed, and color-coded annotations were then ascribed.

A representative of each of the color-coded GnRHR-like clades was subjected to CrustyBase expression pattern analysis. That is, a tBLASTN search of the aa was conducted for the following four molt-related transcriptomes: multiple tissues, 11 stages of embryo development, 12 stages of larval development of the ornate spiny lobster (*P. ornatus*), and Y-organ across five molt stages of the blackback land crab (*G. lateralis*). The contigs with E-values less than approximately 1e-50 were all downloaded. The GPCR topological domains (predicted using DeepTMHMM) and conserved GPCR motifs (DRY, CWxP and NPxxY) were sequenced for these contigs to determine which clade they actually represented. This is, as opposed to just returning the lowest E-value in the tBLASTN search. The expression patterns of the legitimized contigs were then compiled for each of the transcriptome contexts and are presented in the results.

## 3 Results

### 3.1 Catalogue of GPCRome sequences

The class A and class B GPCR-encoding transcripts of *P. clarkii*, *H. americanus*, *S. verreauxi*, *Cancer borealis*, *N. norvegicus*, *C. maenas*, *G. lateralis*, and *P. monodon*, whose aa sequences have been predicted previously by the authors and others, were subjected to contemporary workflows and compiled in one convenient spreadsheet (retaining original names, as applicable). We also present the novel GPCR-encoding transcripts of *P. ornatus*, *C. quadricarinatus* and *E. sinensis* (see **Supplementary Material S1**).

### 3.2 Clustering analysis and determination of GPCR topological domains

**Figure 1:**
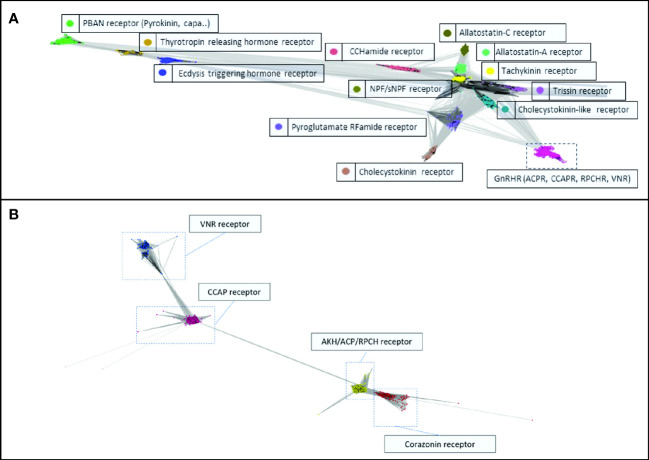
Cluster analysis of **(A)** the class A GPCR-encoding transcripts, and **(B)** the Gonadotropin-Releasing Hormone (GnRH) superfamily of receptor-encoding transcripts with predicted ligands. The images were generated using the CLANS2 algorithm with E-values less than 1e-50.

Transcript ORFs that clustered strongly with the seeded transcripts of predicted receptors of the GnRH superfamily of neuropeptides (that is, displaying E-values less than 1e-50) were identified and extracted (see **Supplementary Material S1**). **Figure 1** depicts the clustering of the class A receptor transcripts (**Figure 1A**) and the constituent members of the GnRHR-like superfamily (**Figure 1B**). The topological GPCR domains of the GnRHR-like predicted ORFs, including the N-terminus and C-terminus, ICLs and ECLs, and the canonical 7TM domains have been predicted and are presented in **Supplementary Material S1**. DRY, CWxP, and NPxxY motif loci were sequenced for the GnRHR-like transcripts and have also been reported (see **Supplementary Material S1**).

### 3.3 Diversity of DRY motif locus sequences

**Table 2** summarizes the diversity observed in the aa sequences of the function-related DRY motif loci of this dataset of decapod GPCRs and, for comparison, a dataset of human GPCRs analyzed by others (8). Noting the high degree of conservation of the DRY motif, and the similarity between datasets.

**Table 2:** Heatmaps depicting the diversity of the amino acid sequences of GPCR-encoding transcript DRY motif loci of A) the eleven decapod species reported in this study (n=1289), and B) of a dataset of human GPCRs that was reported by others (n=270) (8).

A	Decapod DRY motif locus			B	Human DRY motif locus		
	D	R	Y		D	R	Y
A	0	1	4	A	1	0	1
C	0	0	3	C	0	1	9
D	59	1	0	D	66	0	0
E	26	0	0	E	20	0	0
F	0	0	18	F	1	0	11
G	2	0	0	G	1	0	1
H	2	0	2	H	2	1	4
I	0	0	0	I	0	0	0
K	0	3	0	K	0	1	0
L	0	0	5	L	0	0	1
M	0	0	1	M	0	0	0
N	6	0	2	N	3	0	0
P	0	0	1	P	0	0	0
Q	1	1	0	Q	3	1	0
R	0	94	0	R	0	96	0
S	0	1	0	S	1	0	1
T	2	0	0	T	2	0	0
V	1	0	0	V	1	0	0
W	1	0	4	W	0	0	4
Y	1	0	61	Y	0	0	67

The numbers represent the percentage of amino acid residues observed in that locus.

**Supplementary Material S2**: **Table 1** depicts the diversity of aa residues observed at the DRY motif locus for the 1289 decapod GPCR-encoding transcripts of this study. The conserved DRY motif was observed in 40.8% of the transcripts. Whereas ERY (10.6%), DRF (8.8%), ERF (6.8%), GRF (1.8%), DKY (1.7%), DSY (0.9%), DRC (0.7%), ERC (0.6%), GRY (0.5%), and VRY (0.3%) collectively were observed in 32.7% of the transcripts. Meaning that the variation observed in the DRY motif loci of 73.5% of the transcripts is explained by a point mutation in a single nucleotide of an aa codon. DRY locus motifs containing R as the second aa residue were observed in 93.8% of the transcripts (refer to **Table 2** and the left-hand column of **Supplementary Material S2**: **Table 1**). Interestingly, in the third aa or ‘Y’ locus of the DRY motif, an aromatic aa (that is, W = tryptophan, Y = tyrosine, F= phenylalanine and H= histidine) was observed in 84.5% of the transcripts. Moreover, DRM and GRF motifs were only observed in 0.9% and 1.8% of the GPCR-encoding transcripts, respectively. The clusters of motifs that deviated from the DRY sequence represented in **Supplementary Material S2**: **Table 1** (that is, away from the upper left corner) are noted as being biologically less probable, as is detailed in the accompanying notes (**Supplementary Material S2**). These deviations raise the possibility of function-related consequence if they are not bioinformatic anomalies (for example GRF sequence in the DRY locus).

### 3.4 Conserved motifs of the receptors of the Gonadotropin-Releasing Hormone (GnRH)-like neuropeptides

**Figure 2:**
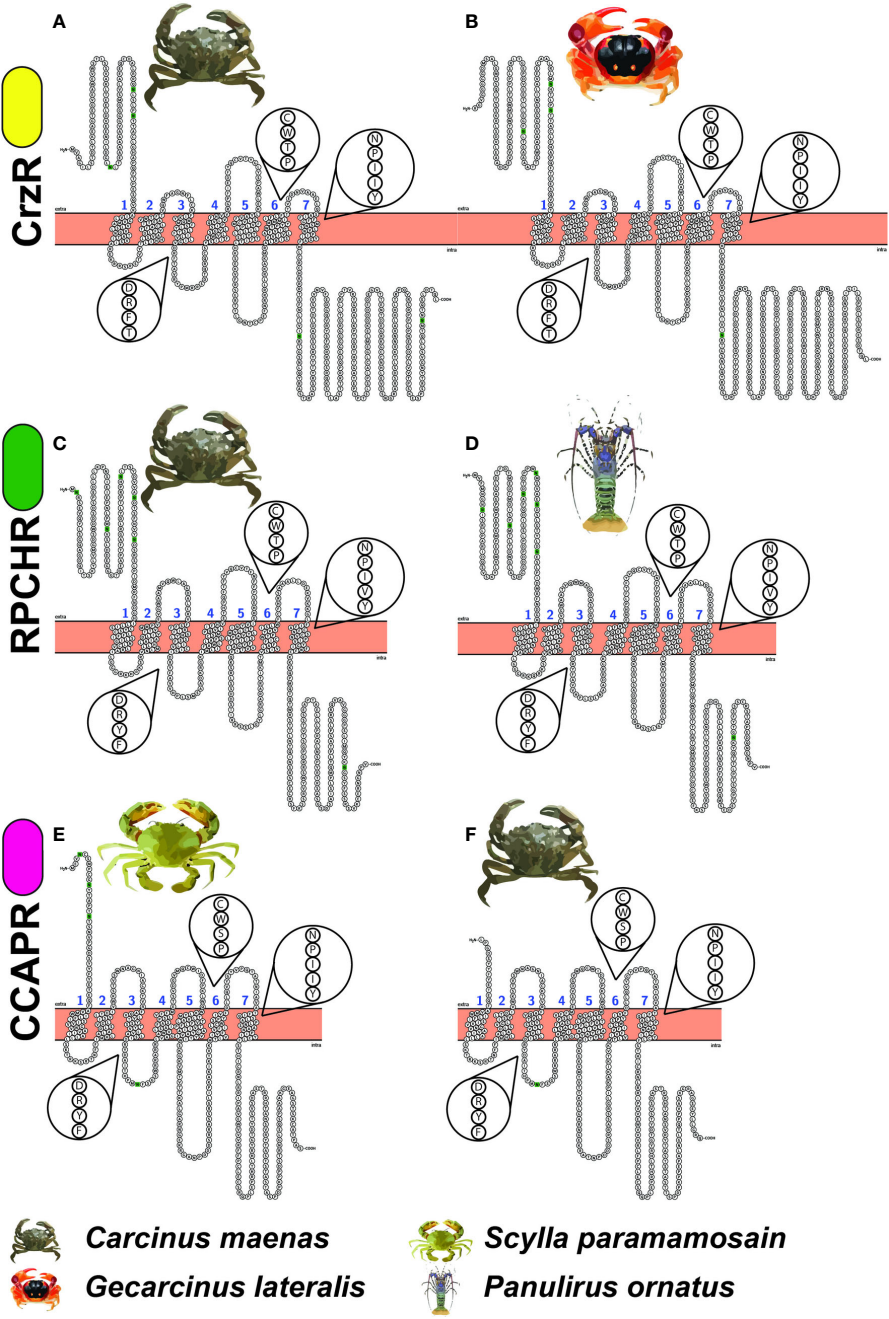
Snake plots of the functionally annotated receptors of **(A)** Corazonin (Cm_CrzR), **(C)** Red Pigment Concentrating Hormone (Cm_RPCHR) and **(E)** Crustacean Cardioactive Peptide (Sp_CCAPR), compared with GPCR-encoding transcripts **(B)** Gl_GPCR_A7 **(D)** Po_GPCR_A146 and **(F)** Cm_GPCR_A117 of this dataset. The exploded views depict the conserved function-related DRY, CWxP and NPxxY motifs. Noting the similarity of the GPCR topological domains that are represented including the N-terminus (H_2_N), C-terminus (COOH), three intracellular loops, three extracellular loops, canonical seven transmembrane domains, N-glycosylation sites (depicted by green) and conserved motifs. Yellow, green and pink ovals mean next to the receptor names correspond to the DRY motifs listed in **Table 3**.

The snake plots in **Figure 2** depict the topological domains of three pairs of GnRHR-like proteins. The receptors on the left-hand side are the functionally annotated receptors of corazonin, RPCH and CCAP. The three receptors on the right-hand side (Gl_GPCR_A7, Po_GPCR_A146 and Cm_GPCR_A117) are transcripts from this dataset that share aa sequence identity with the annotated receptors. Note the high degree of similarity of the N-termini, ECLs, ICLs, C-termini, conserved N-glycosylation sites and conserved motifs (that is, DRY, CWxP and NPxxY). Observed differences in the lengths of N-terminus and C-terminus may be a sequencing artefact.

### 3.5 Relationships between the DRY motifs and the seven transmembrane (7TM) domains of the Gonadotropin-Releasing Hormone (GnRH) superfamily of receptors

**Figure 3:**
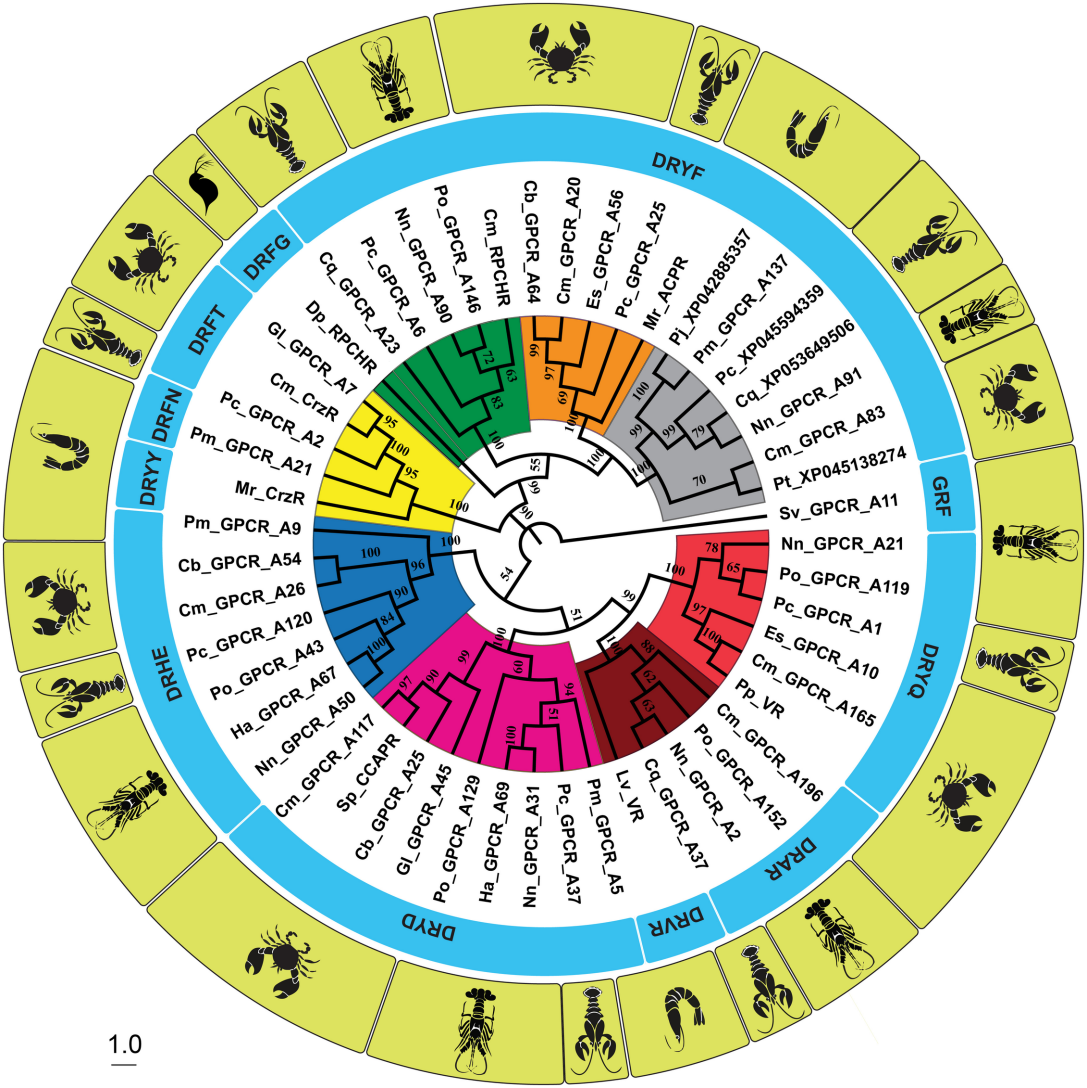
Neighbor-joining cladogram using the seven transmembrane domains, and intracellular and extracellular loops of the Gonadotropin-Releasing Hormone (GnRH) superfamily of receptor transcripts. Labeling of the tree includes the name of the receptor (including the decapod species), the sequence of the functional-relevant ‘DRYx’ motif locus, and an image of the species. The Muscle algorithm was employed in the sequence alignment and bootstrap values of 1000 were used. Legend: *Pc*, *Procambarus clarkii* Louisiana crawfish; *Ha*, *Homarus americanus* American lobster; *Sv*, *Sagmariasus verreauxi* eastern rock lobster; *Cb*, *Cancer borealis* Jonah crab; *Nn*, *Nephrops norvegicus* Norwegian lobster; *Cm*, *Carcinus maenas* Green shore crab; *Gl*, *Gecarcinus lateralis* blackback land crab; *Pm*, *Penaeus monodon* giant tiger prawn; *Po*, *Panulirus ornatus* ornate rock lobster; *Cq*, *Cherax quadricarinatus* Australian red claw crayfish; *Es*, *Eriocheir sinensis* Chinese mitten crab; *Sp*, *Scylla paramamosain* green mud crab; *Pp*, *Portunus pelagicus*blue swimmer crab; *Lv*, *Litopenaeus vannamei*white leg shrimp; *Mr*, *Macrobrachium rosenbergii* giant freshwater prawn; *Pj*, *Penaeus japonicus* kuruma shrimp and *Dp*, *Daphnia pulex* water flea. Distinct clades are color-labeled.

The relationship between the phylogeny of the 7TMs (including ICLs and ECLs) of the GnRHR-like protein transcripts, and the associated sequence of the DRYx motif locus and species is summarized in **Figure 3**. Noting the high degree of compartmentalization of the labels (that is, species and DRY motif sequence), and very high bootstrap values within color-coded clades. Moreover, the receptor pairs depicted in the snake plots of **Figure 2** cluster together.

Referring again to the snake plots (**Figure 2**) and the DRYx motif (**Figure 3**), we present this as evidence that Gl_GPCR_A7, Po_GPCR_A146, and Cm_GPCR_A117 are orthologues of Cm_CrzR, Cm_RPCHR and Sp_CCAPR, respectively. Thereby representing candidates for functional annotation as receptors of corazonin, RPCH, and CCAP, respectively.

### 3.6 Annotation of the decapod GnRHR-like superfamily of receptors

The Neighbor Joining phylogenetic trees of both the 7TMs (including ECLs and ICLs) and of the ORFs of the GnRHR-like receptors (seen in **Figure 3** and **Supplementary Material S2**: **Figure 1**, respectively) are presented for comparison. The eight discernible and color-coded clades were composed of the same constituent receptors and had similar bootstrap values. The yellow, green and magenta clades contained the functionally annotated receptors of corazonin, RPCH and CCAP, respectively. The maroon and light red clades contained the two bioinformatically annotated variants of the VR. The blue clade contains Ha_GPCR_A67 of which GNRH2R (accession number: XP_042226332.1) (38) is a truncated version. Notably, the GnRHRs (blue) were more closely related to the VR/CCAPR clade than the CrzR/ACPR1/ACPR2/RPCHR clade, respectively (albeit with the low bootstrap values of 54 and 34). Snake plots of the ACPR1 (orange clade) and ACPR2 (grey clade) receptors are included in **Supplementary Material S2**: **Figures 2**, **3**, respectively. Comparison and contrast of the topological domains depicted in these figures reveals a high degree of intra-clade similarity, and inter-clade similarity but with a discernible degree of inter-clade difference. For example, the length of ECL3, which may have implications for function and/or ligand binding. The orange clade contained the bioinformatically annotated ACPR1 receptor, Mr_ACPR.

**Table 3** summarizes the canonical conserved motifs (DRY, NPxxY, and CWxP) observed in GnRHR-like transcripts of our dataset and in the aa sequences of GPCRs annotated by others (functionally and bioinformatically). Noting the high degree of conservation, congruence and compartmentalization of sequence motif variants within clades of the same predicted ligand, and by extension, the same predicted function. Members of the GnRHR clade (blue) were observed to possess a distinctive DRHEAV sequence in the DRYxxx motif, and lack any discernible CWxP motif, which was exceptional in the dataset tested. Further we noted that tyrosine (Y) is aromatic, hydrophobic and non-charged, whereas histidine (H) is aromatic, hydrophobic and positively charged, which likely has implications for functionality. Altogether, suggesting that the GnRHR receptors (blue) represent a putative novel GPCR model. Moreover, as new models they will likely need novel bioassays for functional annotation. Considering the ACPR clades (orange and grey), the silkworm moth *B. mori* ACPR-like BNGR-A28 and BNGR-A29 had conserved DRYxxx, CWxP and NPxxY motifs of DRFFAI, CWFP and DPLVY, and DRFFAV, CWLP and NPLVY, respectively. Noting a, not unexpected, lack of total congruence between the conserved motifs of the two ACP receptors of *B. mori* (Taxonomical order: Lepidoptera) and the decapods of this study (Taxonomical order: Crustacea) (see **Table 3**). Considering all of the above, this suggests that the yellow, green, magenta, maroon, light red, blue, orange and grey clades represent CrzR, RPCHR, CCAPR, VR1, VR2, and the newly annotated GnRHR, ACPR1 and ACPR2, respectively.

**Table 3:** Conserved GPCR motifs (that is, DRYxxx, CWxP and NPxxY) observed in transcripts of the GnRHR-like receptors of this dataset and in protein sequences published on NCBI.

Name	DRYxxx	CWxP	NPxxY	Annotation	Legend
Pm_GPCR_A9	DRHEAV	TNTPYV	NPFIY	GnRHR	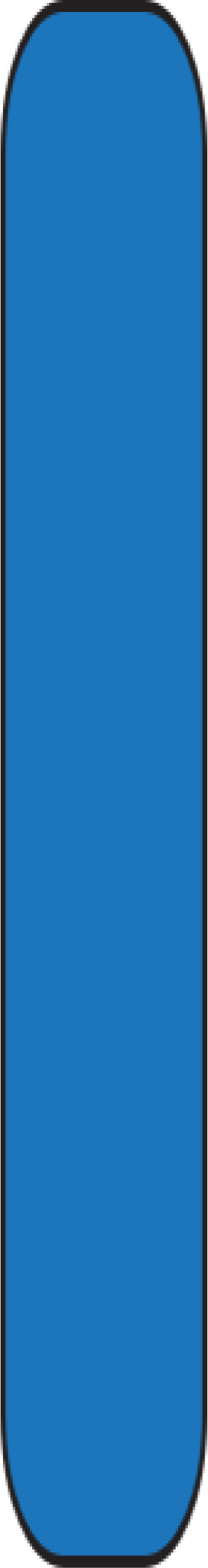
Cb_GPCR_A54	DRHEAV	TNTPYV	NPFIY	GnRHR	
Cm_GPCR_A26	DRHEAV	TNTPYV	NPFIY	GnRHR	
Pc_GPCR_A120	DRHEAV	TNTPYV	NPFIY	GnRHR	
Po_GPCR_A43	DRHEAV	TNTPYV	NPFIY	GnRHR	
Ha_GPCR_A67	DRHEAV	TNTPYV	NPFIY	GnRHR	
Nn_GPCR_A50	DRHEAV	TNTPYV	NPFIY	GnRHR	
Ha_XP042226332	DRHEAV	TNTPYV	NPFIY	GnRHR^1^	
Pm_GPCR_A21	DRFNAV	CWTP	NPIIY	CrzR	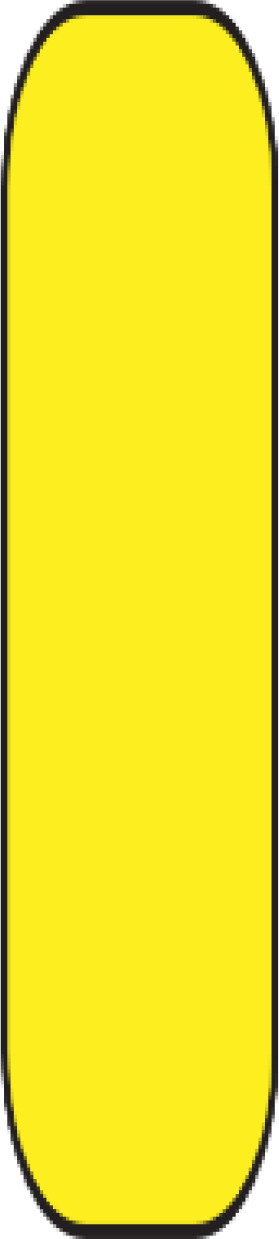
Gl_GPCR_A7	DRFTAV	CWTP	NPIIY	CrzR	
Cm_CrzR	DRFTAV	CWTP	NPIIY	CrzR^2^	
Pc_GPCR_A2	DRFTAV	CWTP	NPLIY	CrzR	
Mr_CrzR	DRYYAV	CWTP	NPIIY	CrzR	
Dp_RPCHR	DRFGAI	CWTP	NPMVY	RPCHR^2^	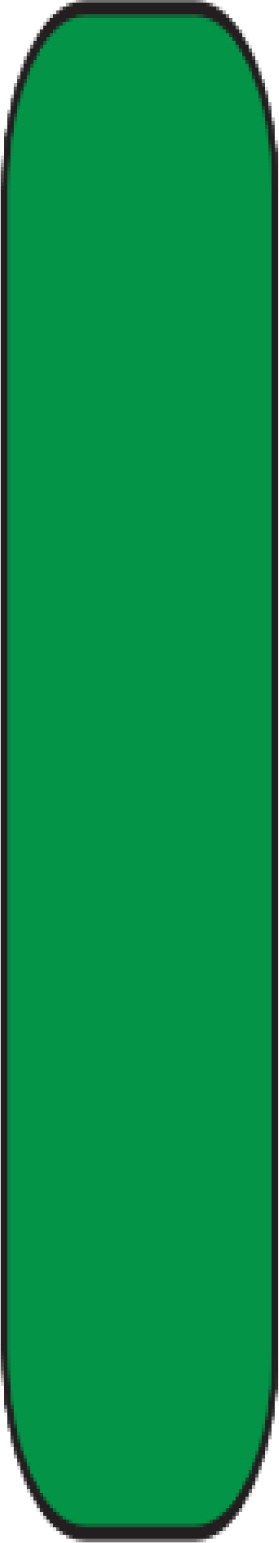
Cq_GPCR_A23	DRYFAV	CWTP	NPIVY	RPCHR	
Pc_GPCR_A6	DRYFAV	CWTP	NPIVY	RPCHR	
Nn_GPCR_A90	DRYFAV	CWTP	NPIVY	RPCHR	
Po_GPCR_A146	DRYFAV	CWTP	NPIVY	RPCHR	
Cm_RPCHR	DRYFAV	CWTP	NPIVY	RPCHR^2^	
Cm_GPCR_A20	DRYFAV	CWTP	NPIIY	ACPR1	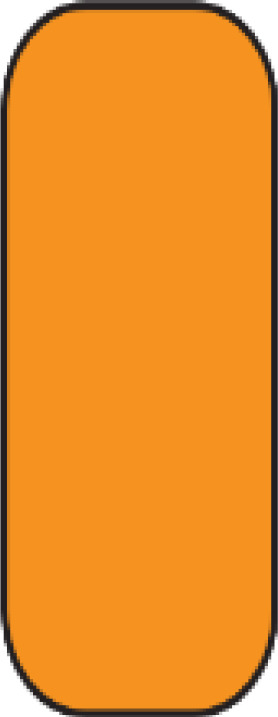
Cb_GPCR_A64	DRYFAV	CWTP	NPIIY	ACPR1	
Es_GPCR_A56	DRYFAV	CWTP	NPIIY	ACPR1	
Pc_GPCR_A25	DRYFAV	CWTP	NPIIY	ACPR1	
Mr_ACPR	DRYFAV	CWTP	NPIIY	ACPR1^1^	
Nn_GPCR_A91	DRYFAI	CWTP	NPLVY	ACPR2	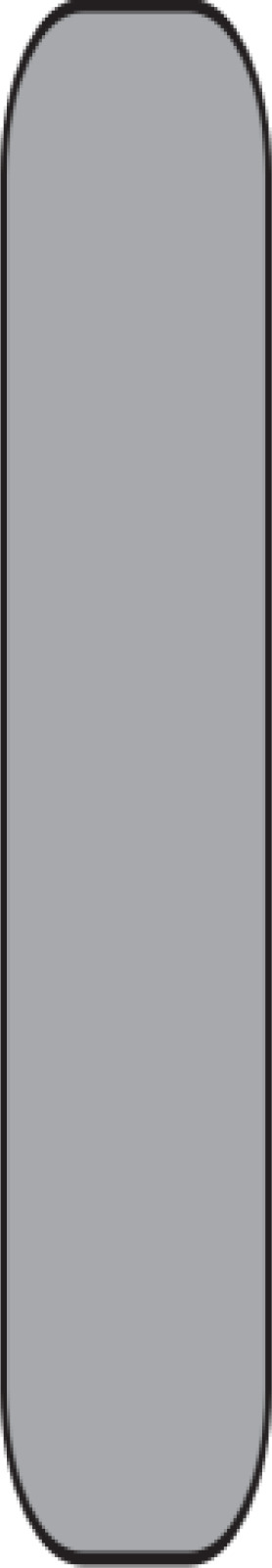
Pm_GPCR_A137	DRYFAI	CWTP	NPLVY	ACPR2	
Cm_GPCR_A83	DRYFAI	CCTP	NPILY	ACPR2	
Pc_XP045594359	DRYFAI	CWTP	NPLVY	ACPR2	
Pt_XP045138274	DRYFAI	CWTP	NPIVY	ACPR2	
Pj_XP042885357	DRYFAI	CWTP	NPLVY	ACPR2	
Cq_XP053649506	DRYFAI	CWTP	NPLVY	ACPR2	
Cm_GPCR_A196	DRARAV	CWSP	NPWIY	VR1	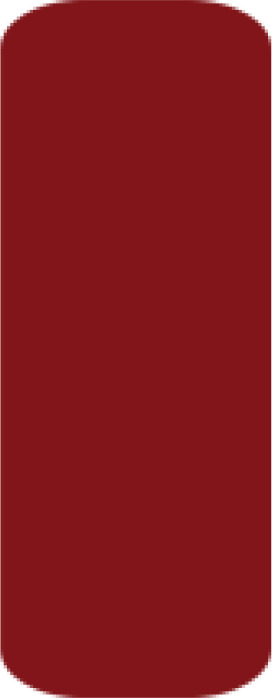
Cq_GPCR_A37	DRARAV	CWAP	Truncated	VR1	
Po_GPCR_A152	DRARAV	CWSP	NPWIY	VR1	
Nn_GPCR_A2	DRARAV	CWAP	NPWIY	VR1	
Lv_VR	DRVRAV	CWSP	NPWIY	VR1	
Po_GPCR_A119	DRYQVI	CSAP	NPWIF	VR2	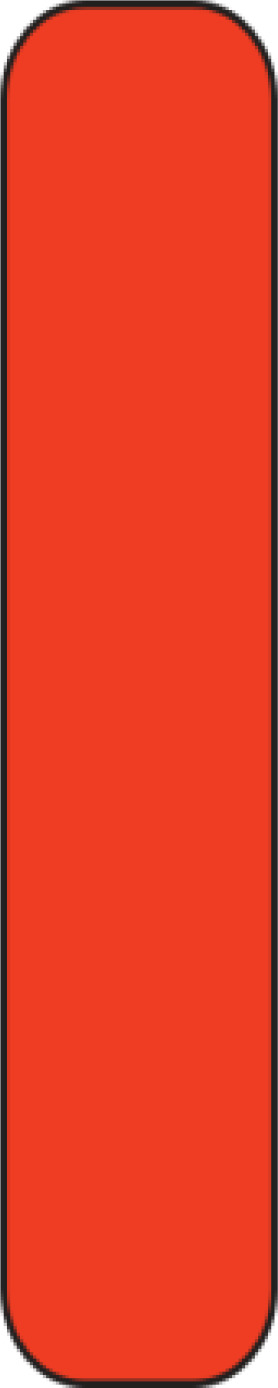
Pc_GPCR_A1	DRYQVI	Truncated	Truncated	VR2	
Cm_GPCR_A165	DRYQVI	CSAP	NPWIF	VR2	
Es_GPCR_A10	DRYQVI	CSAP	NPWIF	VR2	
Pp_VR	DRYQVI	CSAP	NPWIF	VR2	
Nn_GPCR_A21	DRYQVI	CSAP	NPWIF	VR2	
Cm_GPCR_A117	DRYDAI	CWSP	NPIIY	CCAPR	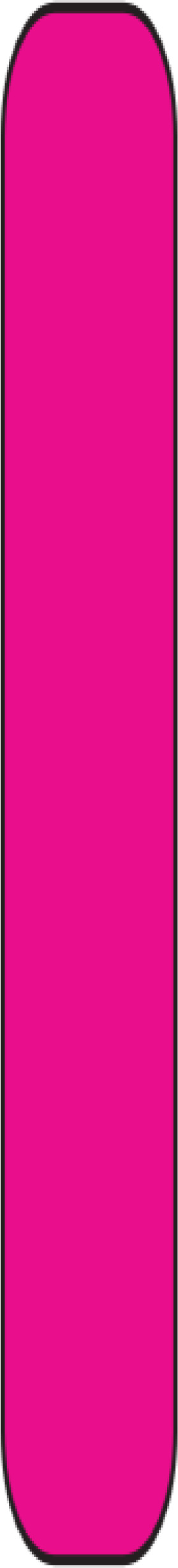
Gl_GPCR_A45	DRYDAI	CWSP	NPIIY	CCAPR	
Ha_GPCR_A69	DRYDAI	CWSP	NPIIY	CCAPR	
Nn_GPCR_A31	DRYDAI	CWSP	NPIIY	CCAPR	
Pc_GPCR_A37	DRYDAI	CWSP	NPIIY	CCAPR	
Pm_GPCR_A5	DRYDAI	CWSP	NPIIY	CCAPR	
Po_GPCR_A129	DRYDAI	CWSP	NPIIY	CCAPR	
Cb_GPCR_A25	DRYDAI	CWSP	NPIIY	CCAPR	
Sp_CCAPR	DRYDAI	CWSP	NPIIY	CCAPR^2^	
Sv_GPCR_A11	GRFVAV	CWTP	NPIIY	Outgroup	

1. Bioinformatically annotated 2. Functionally annotated. 3. Color coding corresponds to the clades observed in the accompanying phylogenetic tree of **Figure 3**. Legend: Pc, Procambarus clarkii Louisiana crawfish; Ha, Homarus americanus American lobster; Sv, Sagmariasus verreauxi eastern rock lobster; Cb, Cancer borealis Jonah crab; Nn, Nephrops norvegicus Norwegian lobster; Cm, Carcinus maenas Green shore crab; Gl, Gecarcinus lateralis blackback land crab; Pm, Penaeus monodon giant tiger prawn; Po, Panulirus ornatus ornate rock lobster; Cq, Cherax quadricarinatus Australian red claw crayfish; Es, Eriocheir sinensis Chinese mitten crab; Sp, Scylla paramamosain green mud crab; Pp, Portunus pelagicus blue swimmer crab; Lv, Litopenaeus vannamei white leg shrimp; Pj, Penaeus japonicus kuruma shrimp; Mr, Macrobrachium rosenbergii giant freshwater prawn; and Dp, Daphnia pulex water flea.

**Figure 4:**
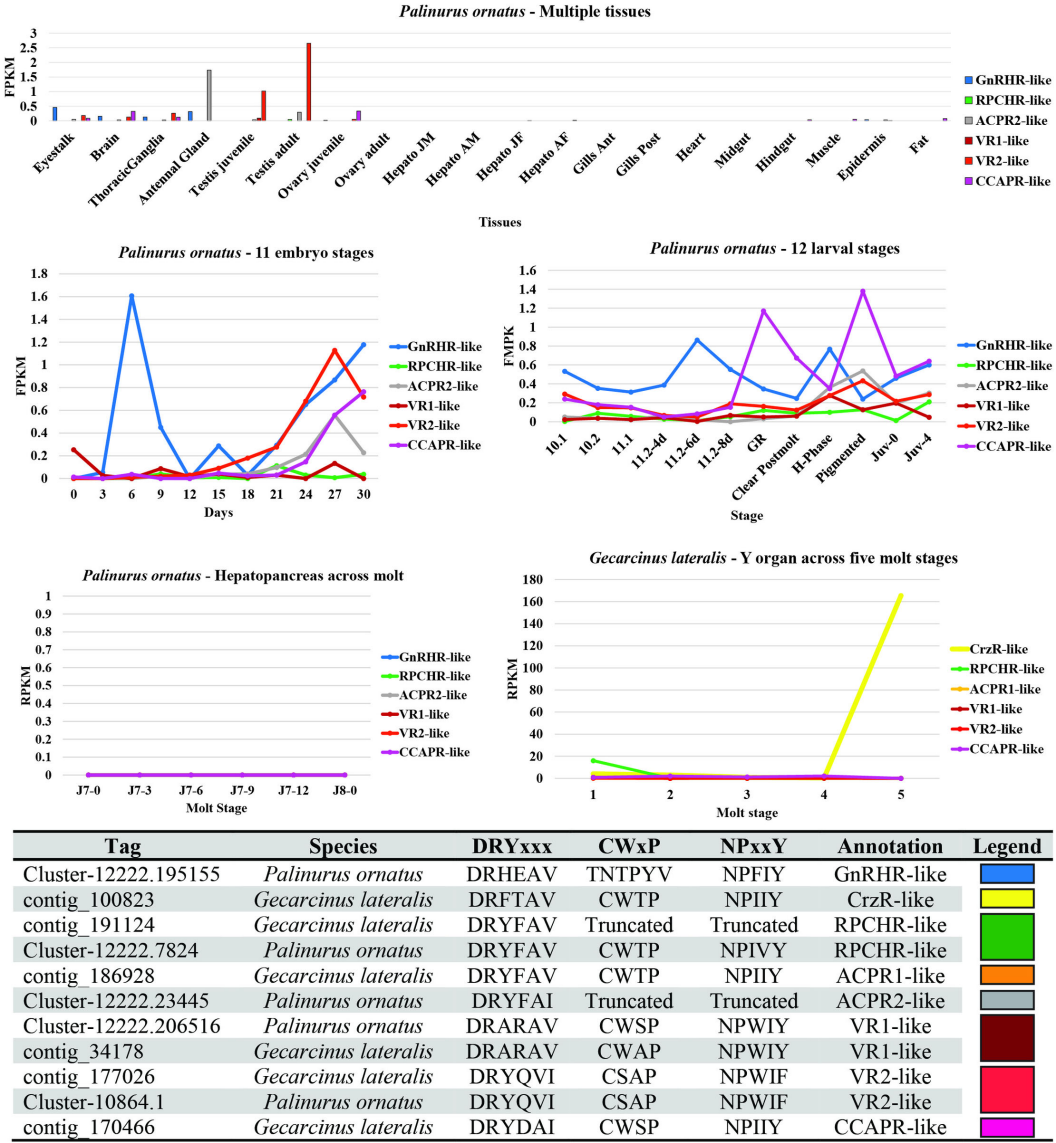
Transcriptomic-related expression patterns of decapod GnRHR-like receptors using the CrustyBase database (39). Tag numbers represent CrustyBase designation.

**Figure 4** summarizes the findings of the CrustyBase expression pattern analysis of the GnRHR-like transcripts. In general, across tissues of *P. ornatus*, gene expression levels were low, however, the highest expression was in neural tissues (eyestalks, brain, thoracic ganglia), antennal gland, and the adult testis. Higher expression of GnRHR-like and CCAPR-like were observed throughout *Po* embryo and larval developmental stages, respectively. Notably the expression of CrzR-like was elevated over the molt stages of *G. lateralis*. ACPR2 (grey) and VR2 (light red) were observed to be expressed in the adult testis, suggesting putative reproduction-related function.

## 4 Discussion

We present several key findings in our study of the GPCRs of decapod species. Foundationally, the GPCRomes of eleven decapod species have been compiled into one convenient source, which also includes the novel GPCR repertoires of three species. We have also developed a novel bioinformatic workflow to augment traditional methods of annotating GPCRs, whose application extends beyond the decapod order. The catalogue of GPCRomes established in this study is a significant achievement in the field of decapod biology for several reasons. Firstly, it is the largest collection of its kind to have been published thus far, comprising a large-scale dataset of GPCR aa sequences. Despite its size, the catalogue is organized in a way that makes it both manageable and easily augmentable (**Supplementary File S1**), ensuring that it remains a valuable resource for researchers over time. Additionally, the inclusion of transcripts derived from a diversity of both species and transcriptomic resources provides valuable insights into the species-specificity of GPCR domains and motifs, rendering it an indispensable tool for the analysis of GPCR amino acid sequences in decapod species.

Our novel bioinformatic workflow is summarized in the following steps. A non-redundant and exhaustive dataset of decapod class A GPCR transcripts was compiled. Clustering analysis was performed using the aa sequences of annotated GPCRs as seeds. Strongly clustering transcripts were extracted, curated for bioinformatic integrity, subjected to a GPCR domain predicting algorithm, and conserved motifs were sequenced. The phylogeny of the 7TM-spanning domains was constructed and labeled with species and conserved DRY motif. Congruence and compartmentalization of the species and conserved motifs was tabulated. All of which represents a superior method of annotating GPCRs than a traditional phylogenetic and BLAST analysis. More specifically, we communicate an absence of the highly conserved CWxP motif in the clade of GnRHR receptors, which was unique among GnRHR-like receptors.

At a time when then the description of the evolutionary history of invertebrate GnRHR-like proteins is in need of novel methods and tools (5), we present our contribution that is consistent with the GnRHR nomenclature proposed by Zandawala et al. (11). We have constructed phylogenies for the GnRHR-like transcripts based on both the 7TM-spanning domains and the entire ORFs. From which we observed congruence between the clades (and relative bootstrap values) that were formed using both methods, suggesting that either method has utility in the analysis of the evolutionary history of decapod GPCRs. Consequently, we have chosen to use the 7TM-spanning domains with their higher degree of sequence conservation when constructing our phylogenies. However, we advocate that the consideration of the sequences of the N-terminus and C-terminus are crucial to the understanding of the function (that is, ligand binding and G protein interaction, respectively) of GPCRs. Our communicated phylogenies contribute to the resolution of decapod and invertebrate GnRHR-like receptors. Further research will address the G proteins involved in GnRH-like receptor signaling.

Several of the factors that influence the transcriptomic analysis described here include the species, spatiotemporal circumstance, life cycle stage and environmental conditions of the subject. Our dataset is large-scale and diverse, however, larger datasets of greater diversity only stand to strengthen future evaluation of the presence and absence of GPCR-encoding transcripts which is crucial in the solidification of our understanding of species-specific variations. Furthermore, the classical nomenclature for annotating GPCRs is heavily biased toward the binding of a single ligand neuropeptide to its single cognate GPCR receptor with unmistakably high affinity. This has proven adequate for the deorphanisation of a multitude of GPCRs, but proves challenging, in the annotation of the GnRHR-like superfamily of receptors. This is because the neuropeptides of this arthropod superfamily are known to display promiscuous binding with the consequence of pleiotropic and overlapping biological effects (37, 40). With this in mind, we have used color-coding of the clades as an intermediate nomenclature that facilitates seamless communication between this and future datasets while the interpretation of the non-singular relationships of the GnRH-like neuropeptides and GPCRs is strengthened. We contend that this study has produced a readily extensible dataset and future-compatible workflow that constitute a tractable prototyping tool in the future functional annotation of decapod GPCRs.

## 5 Conclusions

In this study we have compiled the most comprehensive publicly available catalogue of decapod GPCR-encoding transcripts, to date. We have developed a novel workflow that includes clustering analysis to determine superfamilies of GPCRs (for example, GnRHR-like receptors), sequencing of the membrane-related domains and conserved function-related motifs, phylogenetic analysis of the seven transmembrane-spanning domains with species and conserved motif labels, and visual representation of the conservation of (that is, congruence and compartmentalization) of the domains and motifs. We have constructed well-resolved phylogenies and noted a high degree of conservation of the sequences and topology of the domains and motifs. This conservation has allowed the identification of species-specific variation (especially in the extracellular loops) that is thought to be influential to ligand binding and function. Our results contribute to the resolution of the evolutionary history of invertebrate GnRHRs, and inform the design of bioassays in the deorphanisation and functional annotation of GPCRs.

## Data availability statement

The datasets presented in this study can be found in online repositories. The names of the repository/repositories and accession number(s) can be found in the article/**Supplementary Material**.

## Ethics statement

The manuscript presents research on animals that do not require ethical approval for their study.

## Author contributions

SB: Conceptualization, Data curation, Investigation, Methodology, Visualization, Writing – original draft. TVN: Conceptualization, Data curation, Investigation, Software, Visualization, Writing – review & editing. SC: Writing – review & editing. AE: Writing – review & editing. QF: Funding acquisition, Writing – review & editing. GS: Funding acquisition, Writing – review & editing. DM: Funding acquisition, Writing – review & editing. TV: Conceptualization, Data curation, Funding acquisition, Investigation, Methodology, Project administration, Resources, Software, Supervision, Validation, Writing – original draft, Writing – review & editing.

## Supplementary material

Supplementary Material S1 GPCR-encoding transcripts for eleven decapod species, and GnRHR-like and CHHR-like topological domains.

Supplementary Material S2 Analysis of DRY locus sequence diversity, GnRHR-like ORF based phylogeny, Snake plots of ACPR1 and ACPR2.
