# Supplementary material for: Evaluating conserved domains and motifs of decapod gonadotropin-releasing hormone G protein-coupled receptor superfamily
Source: Front Endocrinol (Lausanne). 2024 Feb 20;15:1348465. doi: 10.3389/fendo.2024.1348465 (PMC10912298; doi:10.3389/fendo.2024.1348465)
Supplement: Supplementary Material S2 — Analysis of DRY locus sequence diversity, GnRHR-like ORF based phylogeny, Snake plots of ACPR1 and ACPR2. [file DataSheet_2.docx]

Supplementary Material 2

Decapod G protein-coupled receptors demystified: evaluation of conserved domains and motifs of gonadotropin-releasing hormone receptor superfamily

**Supplementary Table 1**: Heatmap of the amino acid diversity observed at the DRY motif loci of decapod G protein-coupled receptor transcripts (n=1289).

**Notes:** The numbers represent the percentage values. Darker shades of grey are intended to signify biologically less probable mutation events (that is, multiple nucleotide mutations and/ or mutations other than of the third nucleotide in the codon). See below for a more detailed description.

*Towards understanding the sequence diversity of the DRY motif locus (Supplementary Table 1)*

Considering the D locus, mutation from D to glutamate (E), D to glycine (G), and D to valine (V) can be caused by a single transversion point substitution at the third nucleotide locus, a translation point substitution at the second nucleotide locus, and a transversion point substitution at the second nucleotide locus, respectively. Noting also that E is hydrophilic and negatively charged (like D), whereas G and V are hydrophobic and not charged. Glycine is also the smallest amino acid residue. Considering the R locus, mutation from R to serine (S) can be most readily caused by a transversion at the third nucleotide. Whereas, mutation from R to lysine (K), can be caused by a translation at the second nucleotide. Noting that S is hydrophilic and not charged, whereas K is hydrophilic and positively charge (like R). Considering the Y locus, mutation from Y to phenylalanine (F), and Y to cysteine (C) can be caused by a transversion at the second nucleotide, and a translation at the second nucleotide, respectively. Noting also that F is hydrophobic and not charged and C is moderately hydrophobic and not charged. The prevalence of motifs containing R as the second amino acid residue (93.8%), seen in the left-hand column of the heatmap above (Supplementary Data 2: Table 1) suggests an important utility in the function of the DRY locus motif. Important role of R and having an aromatic amino acid in third amino acid locus (that is, F, W, Y or H).


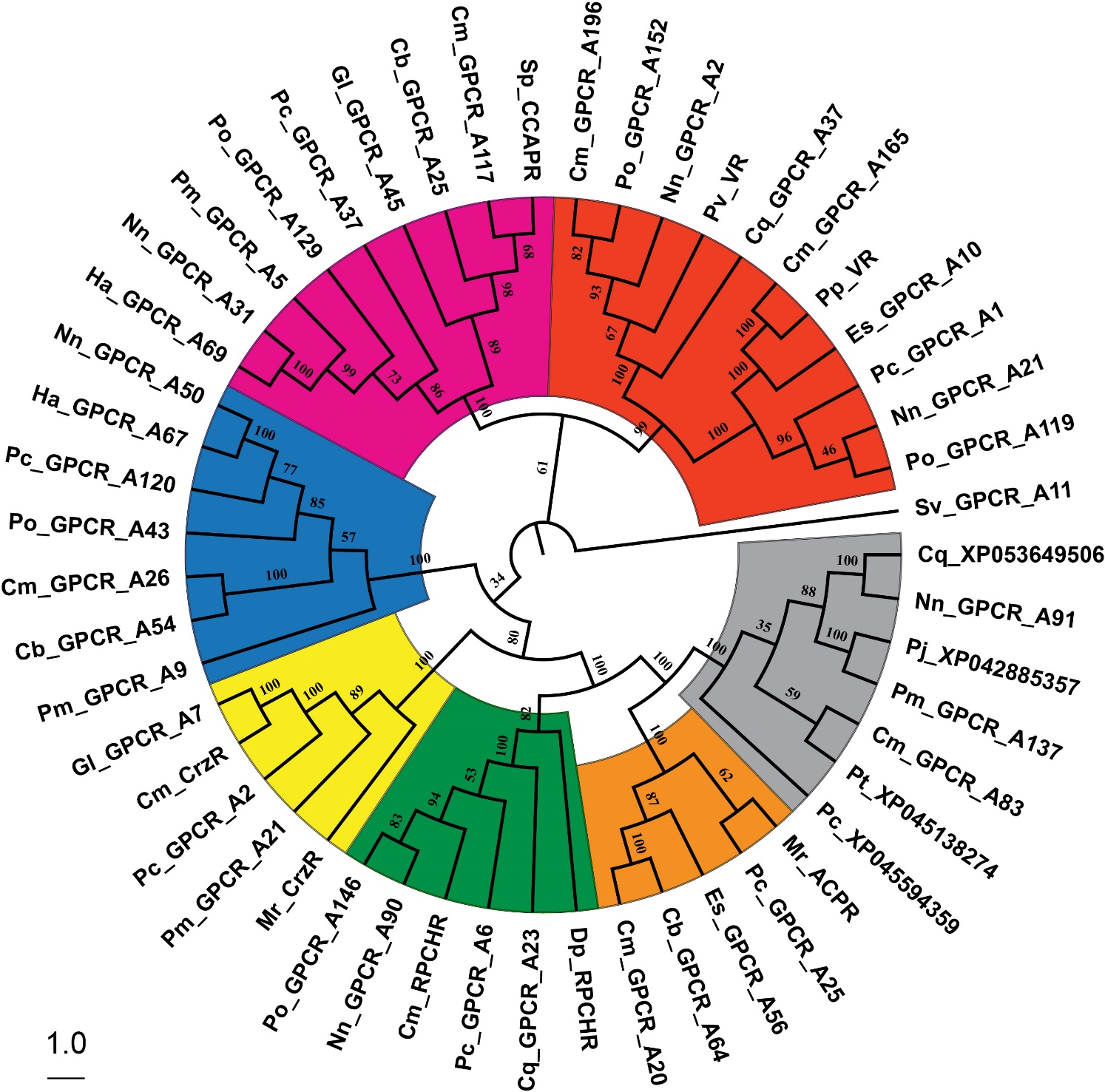


**Supplementary Figure 1:** Neighbor-joining cladogram of the open reading frames (ORFs) of the Gonadotropin-Releasing Hormone (GnRH) superfamily of receptor transcripts with receptor names (including species) as labels. Clear clades are color coded. The Muscle algorithm was employed in the sequence alignment and bootstrap values of 1000 were used. Legend: Pc = *Procambarus clarkii* Louisiana crawfish, Ha = *Homarus americanus* American lobster, Sv = *Sagmariasus verreauxi*eastern rock lobster, Cb = *Cancer borealis* Jonah crab, Nn = *Nephrops norvegicus* Norwegian lobster, Cm = *Carcinus maenas* Green shore crab, Gl = *Gecarcinus lateralis* blackback land crab, Pm = *Penaeus monodon* giant tiger prawn, Po = *Panulirus ornatus* ornate rock lobster, Cq = *Cherax quadricarinatus* Australian red claw crayfish, Es = *Eriocheir sinensis* Chinese mitten crab, Sp = *Scylla paramamosain* green mud crab, Pp = *Portunus pelagicus* blue swimmer crab, Pv = *Penaeus vannamei* whiteleg shrimp, Mr = *Macrobrachium rosenbergii* giant freshwater prawn, Pj = *Penaeus japonicus* kuruma shrimp and Dp = *Daphnia pulex* water flea.


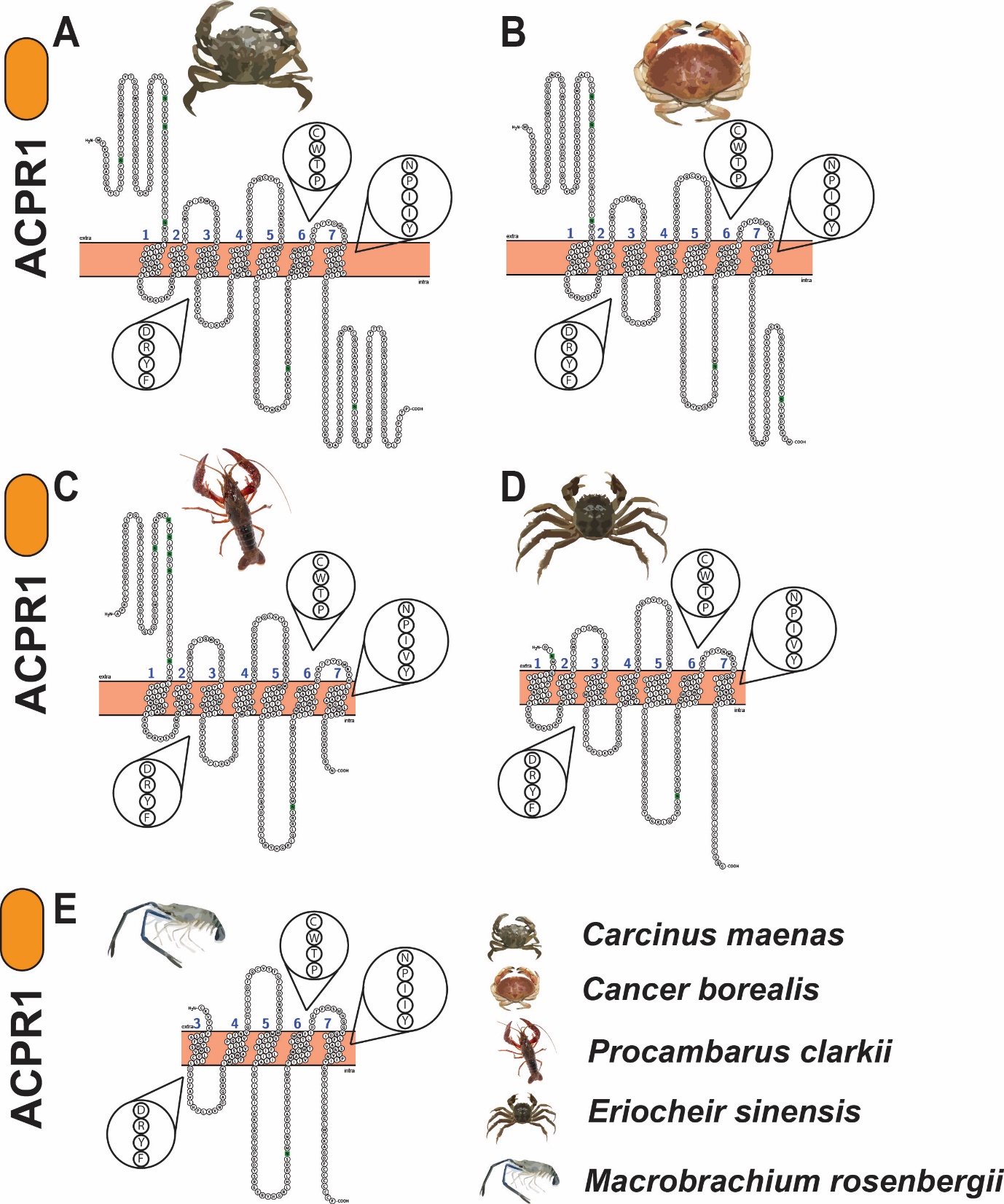


**Supplementary Figure 2:** Snake plots of the ACPR1-like GPCRs: A) Cm_GPCR_a20 B) Cb_GPCR_a64 C) Pc_GPCR_a25 D) Es_GPCR_a56 and E) Mr_ACPR. The magnified views depict the conserved function-related DRYx, CWxP and NPxxY motifs. Noting the similarity of the GPCR topological domains that are represented including the N-terminus (H_2_N), C-terminus (COOH), three intracellular loops, three extracellular loops, canonical seven transmembrane domains, N-glycosylation sites (depicted by green) and conserved motifs.


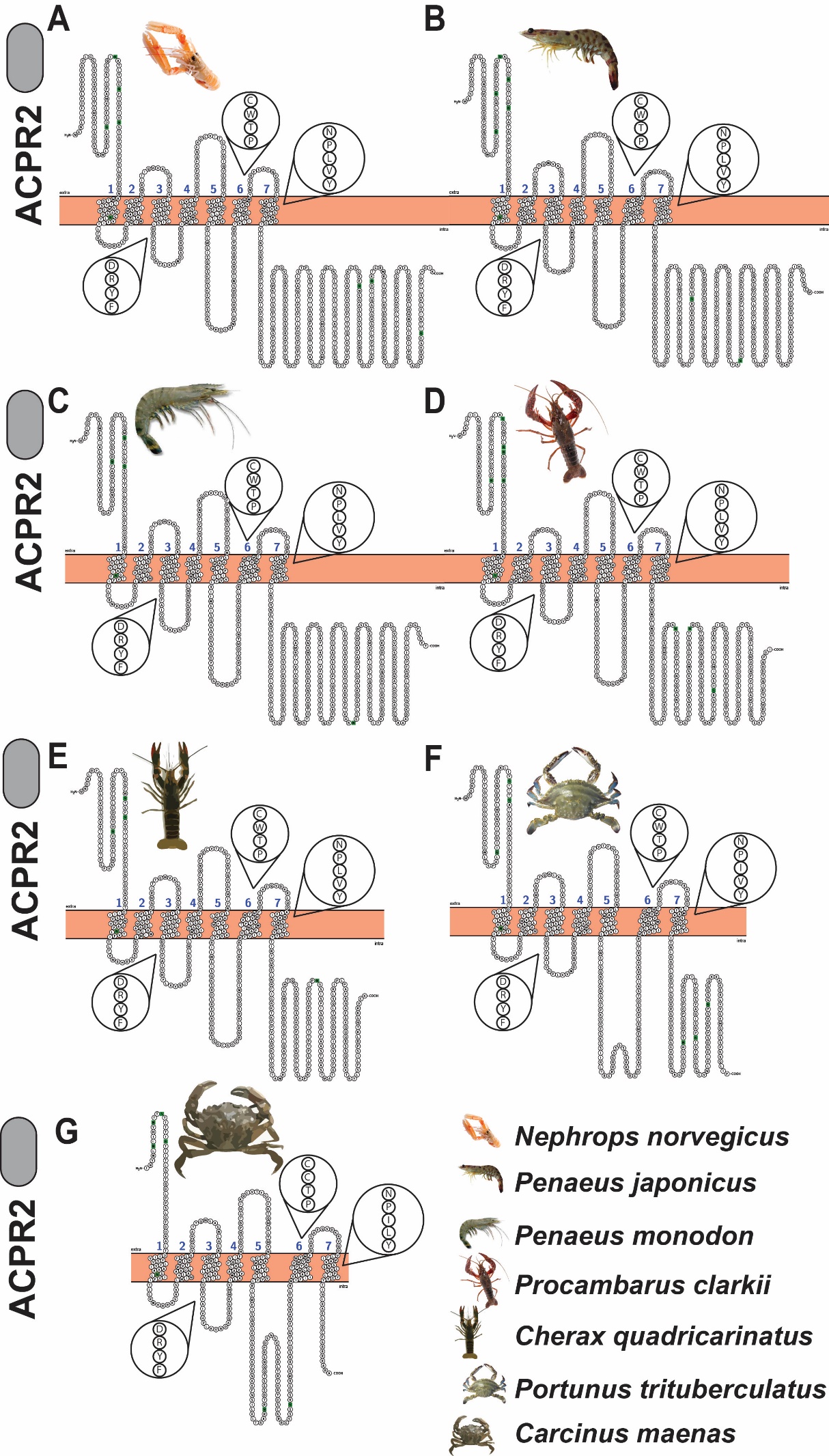


**Supplementary Figure 3:** Snake plots of the ACPR2-like GPCRs: A) Nn_GPCR_A91, B) Pj_XP042885357, C) Pm_GPCR_A137, D) Pc_XP045594359, E) Cq_XP053649506, F) Pt_XP045138274 and G) Cm_GPCR_A83. The magnified views depict the conserved function-related DRYx, CWxP and NPxxY motifs. Noting the similarity of the GPCR topological domains that are represented including the N-terminus (H_2_N), C-terminus (COOH), three intracellular loops, three extracellular loops, canonical seven transmembrane domains, N-glycosylation sites (depicted by green) and conserved motifs.
